# An excess dietary vitamin E concentration does not influence Nrf2 signaling in the liver of rats fed either soybean oil or salmon oil

**DOI:** 10.1186/s12986-017-0225-z

**Published:** 2017-11-16

**Authors:** Klaus Eder, Marina Siebers, Erika Most, Susan Scheibe, Norbert Weissmann, Denise K. Gessner

**Affiliations:** 1 Institute of Animal Nutrition and Nutrition Physiology, Justus-Liebig-Universität Gießen, Heinrich-Buff-Ring 26-32, 35392 Gießen, Germany; 2 Excellence Cluster Cardio-Pulmonary System (ECCPS), Justus-Liebig-Universität Gießen, Aulweg 130, 35392 Gießen, Germany

**Keywords:** Liver, Nuclear factor-erythroid 2-related factor-2, Rat, Unfolded protein response, Vitamin E, Reactive oxygen species

## Abstract

**Background:**

Reactive oxygen species (ROS) are known to stimulate the activation of nuclear factor-erythroid 2-related factor-2 (Nrf2), the key regulator of the antioxidant and cytoprotective defense system in the body. The hypothesis underlying this study was that high dietary concentrations of vitamin E suppress Nrf2 activation, and thus could weaken the body’s antioxidative and cytoprotective capacity. As the effect of vitamin E on Nrf2 pathway might be influenced by concentrations of fatty acids susceptible to oxidation in the diet, we used also diets containing either soybean oil as a reference oil or salmon oil as a source of oil rich in n-3 polyunsatuated fatty acids.

**Methods:**

Seventy-two rats were divided into 6 groups of rats which received diets with either 25, 250 or 2500 mg vitamin E/kg, with either soybean oil or salmon oil as dietary fat sources according to a bi-factorial experimental design. Electron spin resonance spectroscopy was used to determine ROS production in the liver. qPCR analysis and western blot were performed to examine the expression of Nrf2 target genes in the liver of rats.

**Results:**

Rats fed the salmon oil diet with 25 mg vitamin E/kg showed a higher production of ROS in the liver than the 5 other groups of rats which did not differ in ROS production. Relative mRNA concentrations of *NFE2L2* (encoding Nrf2), *KEAP1* and various Nrf2 target genes, protein concentrations of glutathione peroxidase (GPX), heme oxygenase 1 (HO-1), NAD(P)H quinone dehydrogenase 1 (NQO1) and activities of the antioxidant enzymes GPX, superoxide dismutase and catalase were not influenced by the dietary vitamin E concentration. The dietary fat had also less effect on Nrf2 target genes and no effect on protein concentrations of GPX, HO-1, NQO1 and activities of antioxidant enzymes. Dietary vitamin E concentration and type of fat moreover had less effect on mRNA concentrations of genes and concentrations of proteins involved in the unfolded protein response, a pathway which is closely linked with activation of Nrf2.

**Conclusion:**

We conclude that excess dietary concentrations of vitamin E do not suppress Nrf2 signaling, and thus do not weaken the endogenous antioxidant and cytoprotective capacity in the liver of rats.

## Background

Vitamin E is the most important lipid-soluble antioxidant in the animal body. Its major function is to protect cell membranes and lipoproteins against lipid peroxidation by breaking the lipid radical chain reaction. Moreover, vitamin E acts as a direct scavenger of superoxide and hydroxyl radicals. Low concentrations of vitamin E in animal tissues, caused by an insufficient dietary intake, enhance lipid peroxidation which in turn results in the development of oxidative stress. On the opposite, at high tissue concentrations of vitamin E, lipid peroxidation is suppressed and reactive oxygen species (ROS) generated in metabolism are scavenged [1]. High concentrations of ROS in tissues are undoubtedly harmful, leading to oxidative stress, impairment of cellular function and contribute to the development of several diseases [2]. However, physiological concentrations of ROS are acting as important physiological regulators of intracellular pathways [3–7]. Recent studies provided even evidence that a moderate stimulation of the production of ROS in the mitochondria extends the life span in diverse organisms. This phenomenon has been named “mithormesis”. In this context, it has been suggested that oxidants are serving as molecular signals which exert downstream effects to induce endogenous defense mechanism while antioxidant supplements that prevent these oxidant signals interfere with these health-promoting effects [8, 9].

Nuclear factor-erythroid 2-related factor-2 (Nrf2), a redox sensitive transcription factor, is the most important regulator of the antioxidant and cytoprotective defense system in the body [10]. In its inactive state, Nrf2 is sequestered by Keap1, a protein which inhibits the translocation of Nrf2 into the nucleus. ROS produced in the cell are able to react with critical cysteine sulfhydryl groups present in Keap1, leading to its degradation by the ubiquitine proteasome system and activation of Nrf2. The active Nrf2 then translocates into the nucleus and transactivates the expression of a broad spectrum of antioxidant and cytoprotective genes containing an antioxidant response element in their promoters [11]. Thus, Nrf2 protects the body against damage by oxidants and other endogenous or exogenous insults and exerts a preventive effect against various disorders including cancer, heart disease or diabetes [12]. Notably, the beneficial effects of dietary secondary plant metabolites or exercise in the prevention of various diseases including coronary heart disease or diabetes are induced by an activation of Nrf2, which also proves the important role of this transcription factor for human health [13, 14].

Based on the fact that activation of Nrf2 is induced by ROS, a relationship between tissue vitamin E concentrations and the expression of Nrf2 target genes is likely. The existence of such a relationship is supported by the finding that pro-oxidant conditions, such as induced by feeding oxidized fats in animals, are leading to an activation of Nrf2 [15]. On the other side, an excess of dietary vitamin E has been shown to reduce the activity of superoxide dismutase (SOD), a target gene of Nrf2, in human muscle samples and in rat erythrocytes [16, 17]. In the present study, we investigated the hypothesis that feeding diets with excess concentrations of vitamin E could cause an inhibition of the Nrf2 pathway due to a diminishment of the basal levels of free radicals in the cell. For this end, we performed a study with rats as an animal model which received diets with three different levels of vitamin E, either 25, 250 or 2500 mg/kg diet. The lowest dietary vitamin E represents a dose close to the recommendation of vitamin E for rat diets by NRC [18], the two other diets are reflecting conditions with moderate or strong excess of dietary vitamin E. Assuming that the daily organic dry matter intake of human adults is around 400 to 500 g per day, the highest dietary vitamin E concentration of 2500 mg/kg is close to an intake of 1000 mg of vitamin E in human which has been defined as the tolerable upper intake level for vitamin E in adults [19]. As the effect of vitamin E on Nrf2 pathway might be influenced by dietary concentrations of fatty acids susceptible to oxidation, we used also diets containing salmon oil, a source of oil rich in n-3 polyunsaturated fatty acids (PUFA) such as eicosapentaenoic acid (EPA), and docosahexaenoic acid (DHA).

Besides a direct activation by ROS, Nrf2 pathway can also be activated by stress of the endoplasmic reticulum (ER stress). ER stress represents a state of the ER when its function (protein biosynthesis, folding and trafficking, Ca^2+^ homeostasis) is perturbed. ER stress leads to the activation of a network of cytoplasmic and nuclear signaling pathways collectively termed the unfolded protein response (UPR). The initial aim of the UPR is to re-establish ER homeostasis and maintain cell function through inhibition of protein synthesis to decrease ER workload, transcriptional activation of chaperone genes to increase the ER folding capacity, and activation of the ER-associated degradation machinery to clear misfolded proteins [20]. ER stress in the liver is best known from obese animals or animals fed high fat diets [21, 22]. Besides high plasma concentrations of non-esterified fatty acids and inflammation, oxidative stress is regarded as one of the main reasons for the development of ER stress in these animal models [23]. Activation of Nrf2 by UPR has been suggested to be a means to counteract oxidative stress provoked under ER stress conditions [24]. As effects of dietary vitamin E on Nrf2 pathway could be mediated by ER stress, we also considered genes of UPR.

## Methods

### Animal and housing

A total of seventy-two male, 8 week old Sprague-Dawley rats from Harlan (Horst, Netherlands) with an initial body weight of 314 ± 15 g (mean ± SD) g were randomly assigned to six groups of twelve rats each. The rats were kept in pairs in Macrolon cages in a room maintained at 22 ± 1 °C, 50–60% relative humidity and lighting (12/12 h light/dark cycles). All experimental procedures were in strict accordance with the recommendations in the guidelines for the care and use of laboratory animals and the Appendix A of European Convention for the Protection of Vertebrate Animals used for Experimental and other Scientific Purposes. In Accordance with Article 4 par. 3 of the German Animal Welfare Law all animals were humanely killed for scientific purpose approved by the Animal Welfare Officer of the Justus-Liebig-University (JLU No. 438_M).

### Diets and feeding

According to a bifactorial experimental design, six different semi-purified diets were used, differing in their concentrations of vitamin E (25, 250 and 2500 mg vitamin E equivalents/kg diet) and source of dietary fat (soybean oil, salmon oil). The basal semi-purified diet consisted of (in g/kg diet) corn starch (552), casein (200), saccharose (100), oil (50), cellulose (50), vitamin and mineral premix (45), L-cysteine (3). Vitamins and minerals, with the exception of vitamin E, were supplemented according to the American Institute of Nutrition (AIN)-93G recommendations for growing rats [25]. The fatty acid composition of soybean oil (obtained from a local supermarket) and salmon oil (obtained from Caelo GmbH, Hilden, Germany) is shown in Table 1. To adjust the vitamin E concentrations of the diets, the native concentrations of tocopherols in the oils were analyzed (Table 1). Concentrations of α-tocopherol equivalents in the oils were calculated by assuming that the biological activities of γ- and δ-tocopherols are 10% and 3% of α-tocopherol, respectively [26]. Based on the native concentrations of α-tocopherol equivalents of the oils, the diets were supplemented individually with all-rac-α-tocopheryl acetate, considering that the biopotency of all-rac-α-tocopheryl acetate is 67% of α-tocopherol. Diets were prepared by mixing the dry components and subsequent pelleting with a standard pelleting device (Kahl Laborpressanlage Typ 14–175; Reinbek, Germany). The diets were administered ad libitum for a period of 27 days, and food intake was recorded weekly. The relatively short feeding period of four weeks was selected as studies in literature have shown that liver tocopherol concentrations are strongly influenced by the dietary vitamin E concentration during this period or even shorter [27, 28]. Water was available ad libitum from nipple drinkers during the whole experiment.

**Table 1:** Fatty acid composition and tocopherol concentrations of the dietary fats

	Soybean oil	Salmon oil
Fatty acids (g/100 g total fatty acids)		
14:0	–	6.8
16:0	10.6	13.0
16:1 (n-7)	0.3	6.6
18:0	3.7	2.6
18:1 (n-7 + n-9)	17.9	21.9
18:2 (n-6)	58.6	7.4
18:3 (n-3)	8.6	3.4
20:1 (n-9)	–	2.6
20:4 (n-6)	–	0.9
20:5 (n-3)	–	17.1
22:5 (n-3)	–	1.8
22:6 (n-3)	–	12.6
Tocopherols (mg/kg)		
α-tocopherol	70	95
γ-tocopherol	360	25
δ-tocopherol	51	–
Total tocopherol equivalents	108	98

### Sample collection

At day 28, the rats were decapitated under CO_2_ anesthesia. Blood was collected into EDTA polyethylene tubes (Sarstedt, Nürnbrecht, Germany), and plasma was separated from blood by centrifugation (1100 x g; 10 min) at 4 °C. The liver was excised. Afterwards, plasma and liver samples were stored at −80 °C pending analysis.

### Concentration of Tocopherol

Concentrations of tocopherols in dietary oils and liver were determined by HPLC [29]. Samples of about 0.1 g of oil or liver were mixed with 2 mL of a 0.1 g/L pyrogallol solution (ethanol absolute) and 0.3 mL saturated sodium hydroxide solution. The mixture was heated for 30 min at 70 °C and tocopherols were extracted with 2 mL of n-hexane. Individual tocopherols were separated isocratically using a mixture of methanol and water (96:4, *v*/v) as the mobile phase and a LiChrospher 100 RP18 column (5 μm particle size, 125 mm length, 4.6 mm internal diameter, Merck) and detected by fluorescence (Fluorescence Detector L-7480, Merck-Hitachi; excitation wavelength, 295 nm, emission wavelength, 325 nm). The temperature of the column was set at 40 °C using a column oven (L-7360, Merck-Hitachi).

The fatty acid composition of the experimental oils was determined by gas chromatography. Oils were transmethylated into fatty acid methyl esters (FAME) with trimethylsulfonium hydroxide. FAME were separated by gas chromatography using a Chrompack 9000 system (Waldbronn, Germany) equipped with an automatic split injector, a polar capillary column (60 m FFAP, 0.25 mm internal diameter, 0.25 μm film thickness; Macherey and Nagel, Düren, Germany), and a flame ionization detector. Helium was used as the carrier gas with a flow rate of 1 mL/min. FAME were identified by comparing their retention times with those of individually purified standards [30].

### Antioxidant enzymes and glutathione

Catalase (CAT) activity was determined in liver homogenate at 25 °C using hydrogen peroxide as substrate according to the method of Aebi et al. (1984) [31]. One unit of of CAT activity is defined as the amount consuming 1 μmol hydrogen peroxide/min. Total superoxide dismutase (SOD) activities of liver homogenate were determined according to the method of Marklund and Marklund [32] with pyrogallol as the substrate. One unit of SOD activity is defined as the amount of enzyme required to inhibit the autoxidation of pyrogallol by 50%. Glutathione peroxidase (GPX) activity in liver homogenate was determined with hydrogen peroxide as substrate [33]. One unit of GPX activity is defined as the amount consuming 1 μmol hydrogen peroxide/min. Sample protein concentration was determined according to Lowry et al. [34]. Enzyme activities were expressed per mg of protein.

### RNA isolation and qPCR analysis

For qPCR analysis, total RNA was isolated using TRIzol reagent (Invitrogen, Karlsruhe, Germany) according to the manufacturer’s protocol. Concentration and purity of the total RNA were estimated from the optical density at 260 and 280 nm, respectively (NanoQuant Plate and Infinite 200 M microplate reader, Tecan, Männedorf, Switzerland). The cDNA was generated by reverse transcription of 1.2 μg total RNA as described recently in detail [35]. The relative mRNA expression of genes was measured with a Rotor-Gene Q system (Qiagen, Hilden, Germany) using KAPA™ SYBR® FAST qPCR Mastermix (Peqlab, Erlangen, Germany) and gene-specific primer pairs (Eurofins MWG Operon, Ebersberg, Germany). Ct-values of reference and target genes were obtained using Rotor-Gene Q Software (Qiagen). Reference gene stability across samples was determined by performing GeNorm analysis and determination of relative expression of genes was calculated using GeNorm normalization factor including the three most stable [ATP synthase, H+ transporting, mitochondrial F1 complex, beta polypeptide (*ATP5B*), malate dehydrogenase 1 (*MDH1*), calnexin (*CANX*)] out of six reference genes [36]. Gene-specific primer pairs were designed using Primer3 and BLAST. Sequences of specific primers used for qPCR are shown in Table 2. All reactions were performed using the following thermal cycler conditions: 95 °C for 3 min, followed by 35–40 cycles of a three-step reaction: denaturation at 95 °C for 3 s, annealing at 60 °C for 20 s and extension at 72 °C for 1 s. The reaction was followed by a melting curve from 50 °C to 95 °C in 5 s increments of 1 °C to ensure amplification specificity. PCR products were separated electrophoretically using a 1.5% agarose gel stained with GelRed™ nucleic acid gel stain (Biotium, Hayward, CA, USA) to confirm the specifity and expected size of the PCR products.

**Table 2:** *S*equences of specific primers used for qPCR

Gene^a^	Primer Sequence (5` to 3`)^b^		NCBI Accession number
Reference genes			
*ATP5B*	F: GCACCGTCAGAACTATTGCT	R: GAATTCAGGAGCCTCAGCAT	NM_134364.1
*CANX*	F: CCAGATGCAGATCTGAAGAC	R: CTGGGTCCTCAATTTCACGT	NM_172008.2
*MDH1*	F: CAGACAAAGAAGAGGTTGCC	R: CGTCAGGCAGTTTGTATTGG	NM_033235.2
Target genes			
*ATF4*	F: AACACAGCCCTTCCACCTCC	R: TGCTCAGCCCTCTTCTTCTGG	NM_024403.2
*CAT*	F: GTTCAGCGACCGAGGGATTCC	R: TTCCTGTGCAAGTCTTCCTGCC	NM_012520.2
*DDITS*	F: ACAAGCACCTCCCAAAGCCC	R: TGCTCCTTCTCCTTCATGCGC	NM_001109986.1
*DNAJC3*	F: AAGGGTCTGTCACTGCTTCTCC	R: TCTCTAAGCCTTCCCGAATCTGC	NM_022232.1
*EDEM1*	F: ATCCTCGGGTGAATCTGAAGACG	R: TCATAGAAGGAATCCAGCCCAGC	NM_001305279.1
*GCLM*	F: AGCATCCCTGACATTGAAGCCC	R: GCCAAACCACCACATTCACGC	NM_017305.2
*GPX1*	F: TCAGTTCGGACATCAGGAGAATGG	R: GGATCGTCACTGGGTGCTGG	NM_030826.4
*GSTA1*	F: TGCAGCTGGAGTAGAGTTTG	R: ATGGGCACTTGCTGGAACAT	NM_031509.2
*HERPUD1*	F: ACCGTAGTCATGTACCTGCACC	R: TCAGGGTCCAGCACTTCACG	NM_053523.1
*HMOX1*	F: AGCATGTCCCAGGATTTGTC	R: TCACCAGCTTAAAGCCTTCC	NM_012580.2
*HSPA5*	F: TCAGCCCACCGTAACAATCAAGG	R: TCCTCAGCAAACTTCTCGGCG	NM_013083.2
*KEAP1*	F: CCCTGTGCCTCTATGAGCGT	R: TGCCACTCGTCTCGATCTGG	NM_057152.2
*NFE2L2*	F: CCCAGCACATCCAGACAGACA	R: GGCTGGGAATATCCAGGGCAA	NM_031789.2
*NQO1*	F: CCTGGAAGGGTGGAAGAAGCG	R: ATCTGGTTGTCGGCTGGAATGG	NM_017000.3
*PDIA4*	F: GCAAAGATTGACGCGACCTC	R: GGTGGAGGTGTCCAATCAGG	NM_053849.1
*SOD1*	F: TATGGTGGTCCACGAGAAAC	R: AATCACACCACAAGCCAAGC	NM_017050.1
*SULT1B1*	F: TCGCTGGAAATGTGGCCTATGG	R: TGGGCAGATGGGTGTAATTGACC	NM_022513.2
*UGT1A6*	F: CCCGCTATCGCTCCTTTGGG	R: CAGCCAGGATCACACCACAGG	NM_001039691.2

^a^ *ATP5B* ATP synthase, *H+* transporting, mitochondrial F1 complex, beta polypeptide, *ATF4* activating transcription factor 4, *CANX* calnexin, *CAT* catalase, *DDIT3* DNA damage inducible transcript 3, *DNAJC3* DnaJ heat shock protein family (Hsp40) member C3, *EDEM1* ER degradation enhancing alpha-mannosidase like protein 1, *GCLM* glutamate cysteine ligase, modifier subunit, *GPX1* glutathione peroxidase 1, *GSTA1* glutathione S-transferase alpha 1, *HERPUD1* homocysteine inducible ER protein with ubiquitin like domain 1, *HMOX1* heme oxygenase 1, *HSPA5* heat shock protein family A member 5, *KEAP1* kelch like ECH associated protein 1, *MDH1* malate dehydrogenase 1, *NFE2L2* nuclear factor, erythroid 2 like 2, *NQO1* NAD(P)H quinone dehydrogenase 1, *PDIA4* protein disulfide isomerase family A, member 4, *SOD1* superoxide dismutase 1, *SULT1B1* sulfotransferase family 1B member 1, *UGT1A6* UDP glucuronosyltransferase family 1 member A6

^b^ *F* forward, *R* reverse

### Immunoblotting

Preparation of liver homogenates, determination of the protein concentration in the homogenates, protein separation by 12.5% SDS-PAGE, and transfer to a nitrocellulose membrane were carried out as recently described [37]. Nuclear extracts were prepared using a Nuclear Extract Kit (Active Motif, Rixensart, Belgium) according to the manufacturer’s protocol. Protein concentrations were determined by the bicinchoninic acid protein assay kit (Interchim, Montluçon, France) with BSA as standard. 15 μg protein were separated by 10% SDS-PAGE and electro-transferred to nitrocellulose membrane (Pall Corporation, Pensacola, FL, USA). Reversible Ponceau S (Carl Roth, Karlsruhe, Germany) staining was performed to check equal protein transfer to the membranes. After that, the membranes were washed and blocked for 1 h at room temperature with 5% nonfat dry milk in TBS-T (*w*/*v*) following incubations with primary antibodies against BIP (rabbit polyclonal anti-GRP78 antibody, Thermo Fisher Scientific Cat# PA5–29705, RRID:AB_2547179, Schwerte, Germany), p-eIF2α (rabbit polyclonal anti-pSer51 antibody, Cell Signaling Technology Cat# 9721, RRID:AB_330951, Frankfurt/Main, Germany), eIF2α (rabbit polyclonal anti-eIF2α antibody, Cell Signaling Technology Cat# 9722, RRID:AB_2230924), GPX (rabbit polyclonal anti-GPX antibody, Abcam Cat# ab22604, RRID:AB_2112120, Cambridge, UK), HO1 (rabbit polyclonal anti-HO1; Abcam Cat# ab68477, RRID:AB_11156457), Abcam), NQO1 (goat polyclonal, Santa Cruz Biotechnology Cat# sc-16,464, RRID:AB_2154339, Heidelberg, Germany) and β-actin (mouse monoclonal anti-β-actin, Abcam Cat# ab6276, RRID:AB_2223210, Abcam) or α-tubulin (rabbit monoclonal anti-α-tubulin, Cell Signaling Technology Cat# 2125, RRID:AB_2619646) as a reference protein for adequate normalization. Subsequently, the membranes were washed and incubated at room temperature with a horseradish peroxidase-conjugated secondary polyclonal anti-rabbit-IgG antibody (Sigma-Aldrich Cat# A0545, RRID:AB_257896), anti-mouse-IgG antibody (Abcam Cat# ab6728, RRID:AB_955440) or anti-goat-IgG antibody (Santa Cruz Biotechnology Cat# sc-2020, RRID:AB_631728), respectively, for 1.5 h. The membranes used for detection of p-eIF2α were stripped for 30 min with mild stripping buffer following washing and incubation with anti-eIF2α antibody to detect total eIF2α protein expression on the same membranes. The HRP activity was detected using chemiluminescent reagents (Amersham ECL Select Western Blotting Detection Reagent, GE healthcare, Freiburg, Germany). The signal intensities of specific bands were detected with a Bio-Imaging system (Syngene, Cambridge, UK) and quantified using Syngene GeneTools software. A protein ladder (PageRuler™ Prestained Protein Ladder, Thermo Fisher Scientific) served as a molecular-weight size marker on every membrane.

### Electron spin resonance spectroscopy (ESR)

For the measurement of ROS release, liver tissue samples of 20 mg were homogenized using at 6800 g for 30 s (Precellys 24 homogenisator, Peqlab, Erlangen, Germany). The samples were incubated for 30 min under normal atmospheric conditions with the spin CMH (Noxygen, Elzach, Germany) at a final concentration of 0.5 mM at 37 ° C in Krebs-Henseleit Buffer adjusted to pH 7.4. All samples were immediately frozen in liquid nitrogen and later determined using a X-band (9.65 GHz) ESR device (EMXmicro, Bruker GmbH, Rheinstetten, Germany) in frozen state, using the following parameters: G-factor 2.0063, center field ~3355 G, microwave power of 2.000 mW, receiver gain 50 dB, modulation amplitude 2.999 G.

### Statistics

Data were statistically analyzed by two-way ANOVA, with vitamin E concentration, source of oil and their interaction as factors, using the Minitab Statistical Software (Rel. 13.0, State College, PA, USA). Prior to statistical analysis, data were checked for normal distribution by Shapiro-Wilk test. For significant interactions (*P* < 0.05), means of the six groups were compared by Fisher’s multiple range tests. Means were considered significantly different for *P* < 0.05. Data presented are shown as means ± standard deviation (MW ± SD).

## Results

### Effect of dietary oil and vitamin E concentration on feed intake and growth of the rats

The body weight at the beginning of the study was similar within the six groups of rats. Rats fed the salmon oil diets showed a slightly higher daily feed intake (+4%) during the feeding period than rats fed the soybean oil diet (P < 0.05) and tended to have higher daily body weight gains (+6%, *P* < 0.10) than rats fed the soybean oil diets, independent of the dietary vitamin E concentration (Table 3). Feed/gain ratios as well as the final body weights of the six groups of rats however did not differ (Table 3).

**Table 3:** Body weights, weight gains and diet intake of rats fed diets with either soybean oil or salmon oil with various vitamin E concentrations (25, 250 or 2500 mg/kg)

Oil	Soybean oil	Soybean oil	Soybean oil	Salmon oil	Salmon oil	Salmon oil	ANOVA (P)		
Vitamin E (mg/kg)	25	250	2500	25	250	2500	Vitamin E	Oil	Vitamin E x oil
Initial weight (g)	314 ± 16	314 ± 15	313 ± 14	314 ± 14	313 ± 14	314 ± 14	0.99	0.98	0.99
Final weight (g)	428 ± 25	435 ± 22	434 ± 26	442 ± 21	438 ± 26	440 ± 19	0.96	0.18	0.71
Body weight gain (g/d)	114 ± 22	122 ± 13	120 ± 17	128 ± 13	124 ± 18	126 ± 14	0.91	0.058	0.50
Diet intake (g/d)	24.0 ± 1.3	24.6 ± 1.4	24.9 ± 1.3	25.7 ± 0.9	25.1 ± 1.5	25.2 ± 1.7	0.82	0.008	0.18
Feed/gain ratio (g/g)	5.9 ± 1.6	5.5 ± 0.5	5.7 ± 0.7	5.5 ± 0.6	5.5 ± 0.5	5.4 ± 0.4	0.71	0.25	0.60

Data are presented as means ± SD (*n* = 12/group)

### Effect of dietary oil and vitamin E concentration on total tocopherol concentrations in plasma and liver of rats

Concentrations of total tocopherols in plasma and liver were lowest in the groups of rats fed the low vitamin E (25 mg/kg) diets and showed a dose-dependent increase with rising concentrations of vitamin E in the diet (Table 4).

**Table 4:** Concentrations of total tocopherols in plasma and liver of rats fed diets with either soybean oil or salmon oil with various vitamin E concentrations (25, 250 or 2500 mg/kg)

Oil	Soybean oil	Soybean oil	Soybean oil	Salmon oil	Salmon oil	Salmon oil	ANOVA (P)		
Vitamin E (mg/kg)	25	250	2500	25	250	2500	Vitamin E	Oil	Vitamin E x oil
Total tocopherols									
Plasma (μmol/L)	15 ± 2^a^	35 ± 5^c^	58 ± 7^e^	12 ± 2^a^	28 ± 4^b^	42 ± 6^d^	<0.001	<0.001	<0.001
Liver (nmol/g)	29 ± 4^a^	141 ± 34^b^	1073 ± 470^d^	24 ± 4^a^	101 ± 13^b^	565 ± 124^c^	<0.001	<0.001	<0.001

Data are presented as means ± SD (*n* = 12/group)

^a,b,c,d^ Different superscript letters indicate significant differences (*P < *0.05)

For plasma and liver total tocopherols, there was an interaction between vitamin E and dietary oil (Table 4). The increase in total tocopherol concentration in plasma and liver by rising dietary vitamin E concentration was greater in the rats fed soybean oil than in the rats fed salmon oil (Table 4).

### Effect of dietary oil and vitamin E concentration on ROS production in the liver of rats

**Fig. 1:**
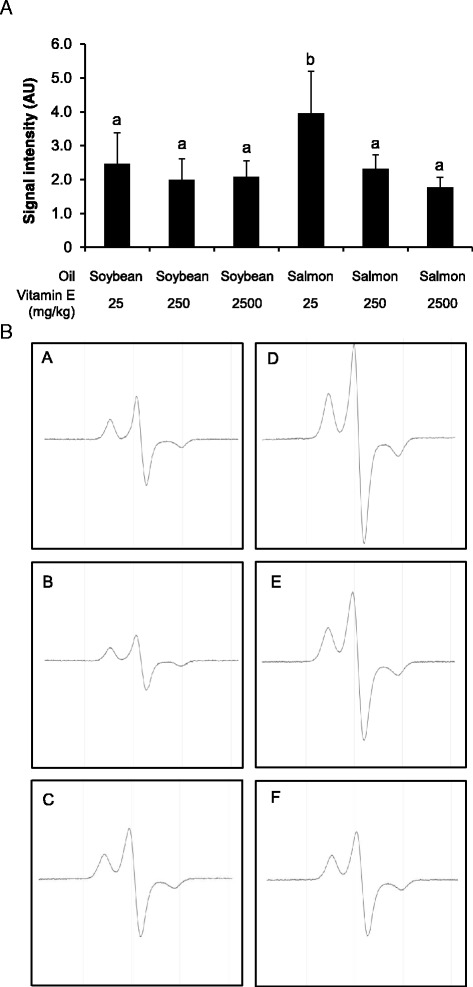
Effect on hepatic ROS production. ESR signal intensity of CMH-detectable ROS in liver of rats fed diets with either soybean oil or salmon oil with various vitamin E concentrations. (A) Bars represent total ROS using the spinprobe molecule CMH and are means ± SD from 8 animals per group; ^a,b^ values with different superscript letters differ significantly (*P* < 0.05). (B) Representative ESR spectra of samples are shown for 1 animal per group (A, diet with soybean oil and 25 vitamin E/kg; B, diet with soybean oil and 250 vitamin E/kg; C, diet with soybean oil and 2500 vitamin E/kg; D, diet with salmon oil and 25 vitamin E/kg, E, diet with salmon oil and 250 mg vitamin E/kg; F, diet with salmon oil and 2500 mg/kg vitamin E)

Rats fed the salmon oil diet with the lowest dietary vitamin E concentration showed the highest production of ROS within liver (Fig. 1). Increasing the dietary vitamin E concentration from 25 to 250 or 2500 mg/kg diet caused a significant reduction of ROS production in the liver of rats fed salmon oil (Fig. 1). In contrast, within the groups of rats fed the soybean oil diet, vitamin E concentration did not influence ROS production in the liver (Fig. 1). Levels of ROS production in the rats fed the soybean oil diets were similar with those observed in rats fed the salmon oil diets with 250 or 2500 mg/kg diet (Fig. 1).

### Effect of dietary oil and vitamin E concentration on hepatic Nrf2 pathway in rats

In order to investigate the effect of dietary treatment on the Nrf2 signaling pathway, we determined relative mRNA concentrations of *NFE2L2* (encoding Nrf2*), KEAP1* and a total of 10 target genes of Nrf2 involved in the antioxidant and drug metabolizing system in the liver. Dietary vitamin E concentration did not influence relative mRNA concentrations of *NFE2L2, KEAP1* and Nrf2 target genes with the only exception of *NQO1* whose relative mRNA concentration was higher in rats fed the diets supplemented with 2500 mg vitamin E/kg diet than in rats fed the diets supplemented with 25 or 250 mg vitamin E/kg diet (Table 5). The dietary oil had a significant effect on relative mRNA concentrations of *NQO1* and *GCLM* (Table 5). Relative mRNA concentrations of these genes were higher in rats fed the fish oil diets than in rats fed the soybean oil diets (*P* < 0.05). Moreover, there were tendencies towards an increase in the mRNA concentrations of *HMOX1* and *SULT1B1* in the rats fed the salmon oil compared to rats fed the soybean oil diet (*P* < 0.10, Table 5). Relative mRNA concentrations of *NFE2L2, KEAP1* and the Nrf2 target genes *SOD1, UGT1A6, GST1A, GPX1* and *CAT* were not influenced by the dietary oil (Table 5). For all the genes considered, there were no interactions between dietary vitamin E concentration and source of oil, meaning that the effects of vitamin E were independent of the source of oil and that the effects of the oil were independent of the dietary vitamin E concentration (Table 5).

**Table 5:** Relative mRNA concentrations of *NFE2L2 (encoding Nrf2), KEAP1 and Nrf2* target genes in the liver of rats fed diets with either soybean oil or salmon oil with various vitamin E concentrations (25, 250 or 2500 mg/kg)

Oil	Soybean oil	Soybean oil	Soybean oil	Salmon oil	Salmon oil	Salmon oil	ANOVA (P)		
Vitamin E (mg/kg)	25	250	2500	25	250	2500	Vitamin E	Oil	Vitamin E x oil
*NFE2L2* ^a^	1.00 ± 0.40	1.02 ± 0.40	0.98 ± 0.55	1.11 ± 0.36	1.11 ± 0.47	1.06 ± 0.34	0.92	0.36	0.99
*KEAP1*	1.00 ± 0.33	1.10 ± 0.62	1.01 ± 0.37	1.05 ± 0.40	1.00 ± 0.53	1.00 ± 0.46	0.94	0.86	0.87
Nrf2 target genes^a^									
*CAT*	1.00 ± 0.18	0.98 ± 0.17	1.02 ± 0.21	1.23 ± 0.25	1.02 ± 0.20	1.14 ± 0.18	0.95	0.29	0.72
*GCLM*	1.00 ± 0.18	1.08 ± 0.15	1.09 ± 0.21	1.18 ± 0.17	1.14 ± 0.19	1.28 ± 0.20	0.18	0.003	0.42
*GPX1*	1.00 ± 0.11	1.13 ± 0.24	1.07 ± 0.26	1.08 ± 0.20	1.13 ± 0.22	1.11 ± 0.23	0.40	0.44	0.86
*GST1A*	1.00 ± 0.23	1.06 ± 0.19	1.06 ± 0.20	1.10 ± 0.23	1.15 ± 0.25	1.11 ± 0.18	0.65	0.16	0.91
*HMOX1*	1.00 ± 0.29	1.07 ± 0.23	1.07 ± 0.32	1.24 ± 0.33	1.24 ± 0.52	1.12 ± 0.29	0.86	0.082	0.65
*NQO1*	1.00 ± 0.20	0.94 ± 0.19	1.23 ± 0.23	1.28 ± 0.27	1.21 ± 0.16	1.50 ± 0.29	< 0.001	< 0.001	0.99
*SOD1*	1.00 ± 0.31	1.18 ± 0.38	1.09 ± 0.36	1.27 ± 0.44	1.17 ± 0.45	1.01 ± 0.29	0.54	0.51	0.28
*SULT1B1*	1.00 ± 0.26	1.00 ± 0.23	1.08 ± 0.18	1.11 ± 0.27	1.22 ± 0.30	1.12 ± 0.27	0.75	0.052	0.52
*UGT1A6*	1.00 ± 0.41	1.11 ± 0.38	1.14 ± 0.29	1.02 ± 0.37	1.00 ± 0.24	0.98 ± 0.20	0.84	0.30	0.66

Data are presented as means ± SD (*n* = 12/group)

^a^ *Abbreviations*: *CAT* catalase, *GCLM* glutamate-cysteine ligase modifier subunit, *GPX1* glutathione peroxidase 1, *GSTA1* glutathione S-transferase alpha 1, *HMOX1* heme oxygenase 1, *KEAP1* kelch like ECH associated protein 1, *NFE2L2* nuclear factor, erythroid 2 like 2, *NQO1* NAD(P)H quinone dehydrogenase 1, *SOD1* superoxide dismutase 1, *SULT1B1* sulfotransferase family 1B member 1, *UGT1A6* UDP glucuronosyltransferase family 1 member A6

**Fig. 2:**
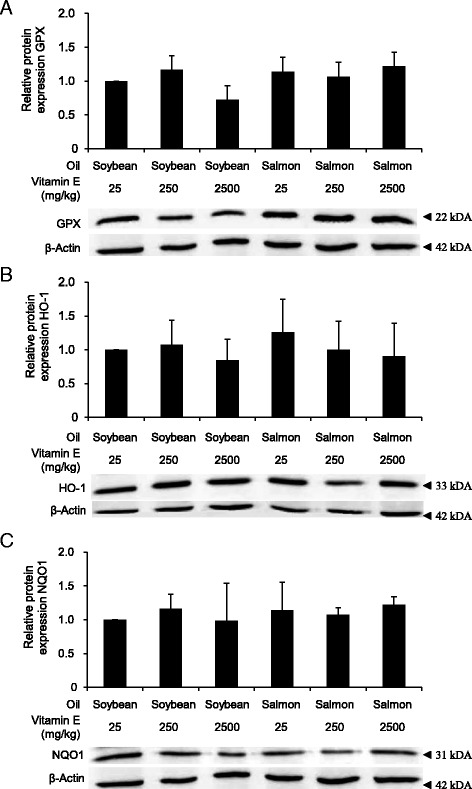
Effect on protein expression of Nrf2 targets. Relative Protein expression of GPX (**a**), HO-1 (**b**) and NQO1 (**c**) in the liver of rats fed diets with either soybean oil or salmon oil with various vitamin E concentrations. Bars represent relative protein level expressed as fold of control (diet with soybean oil and 25 mg/kg vitamin E) and are means ± SD from 12 animals per group. Representative immunoblots specific to GPX (**a**), HO-1 (**b**), NQO1 (**c**) and ß-actin as respective loading control are shown for one animal per group

In order to validate gene expression data on the base of protein concentrations and activity levels, we determined relative protein concentrations of GPX, HO-1 and NQO1, and activities of GPX, SOD and CAT in liver samples. Protein concentrations of GPX, HO-1 and NQO1 and activities of GPX, SOD and CAT were not influenced by the dietary oil and by the dietary vitamin E concentration (Fig. 2, Table 6).

**Table 6:** Activities of glutathione peroxidase (GPX), superoxide dismutase (SOD) and catalase (CAT) in the liver of rats fed diets with either soybean oil or salmon oil with various vitamin E concentrations (25, 250 or 2500 mg/kg)

Oil	Soybean oil	Soybean oil	Soybean oil	Salmon oil	Salmon oil	Salmon oil	ANOVA (P)		
Vitamin E (mg/kg)	25	250	2500	25	250	2500	Vitamin E	Oil	Vitamin E x oil
GPX (U/mg)	1.13 ± 0.26	1.04 ± 0.28	1.02 ± 0.12	1.10 ± 0.29	1.24 ± 0.31	1.01 ± 0.14	0.44	0.27	0.26
SOD (kU/mg)	0.69 ± 0.30	0.87 ± 0.12	0.84 ± 0.29	0.63 ± 0.19	0.84 ± 0.33	0.74 ± 0.28	0.35	0.08	0.88
CAT (kU/mg)	2.14 ± 0.5	2.01 ± 0.58	2.23 ± 0.49	2.32 ± 0.42	2.02 ± 0.54	2.49 ± 0.25	0.24	0.07	0.71

Data are presented as means ± SD (*n* = 12/group)

### Effect of dietary oil and vitamin E concentration on hepatic ER stress pathway in rats

**Fig. 3:**
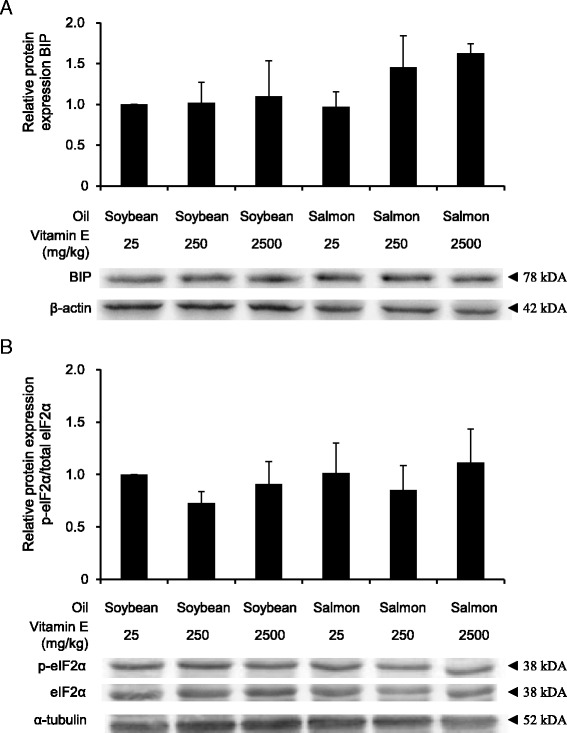
Effect on protein expression of UPR targets. **a** Relative protein expression of BIP and (**b**) ratio of protein expression of p-elF2α and total elf2α in the liver of rats fed diets with either soybean oil or salmon oil with various vitamin E concentrations. Bars represent relative protein level expressed as fold of control (diet with soybean oil and 25 mg/kg vitamin E) and are means ± SD from 12 animals per group. Representative immunoblots specific to BIP (**a**), p-elF2α and total elf2α (**b**) and ß-actin and α-tubulin as respective loading controls are shown for one animal per group

In order to investigate the effect of dietary treatment on ER Stress signaling, we determined relative mRNA concentrations of a total of 8 genes of the UPR pathway in the liver. Dietary vitamin E concentration did not influence relative mRNA concentration of any of the genes considered (Table 6). The dietary oil had no effect on relative mRNA concentrations of all the genes considered with the only exception of *EDEM1* which was lower in the rats fed the salmon oil than in rats fed the soybean oil diet (*P* < 0.05, Table 7). For all the genes considered, there were no interactions between dietary vitamin E concentration and source of oil (Table 7). In order to validate gene expression data on the protein level, we determined relative protein concentration of BIP and the p-EIf2α/total EIf2α ratio by Western Blot. Concentration of BIP and the p-EIf2α/total EIf2α ratio were not influenced by dietary oil and by dietary vitamin E concentration (Fig. 3).

**Table 7:** Relative mRNA concentrations of target genes of the unfolded protein response in the liver of rats fed diets with either soybean oil or salmon oil with various vitamin E concentrations (25, 250 or 2500 mg/kg)

Oil	Soybean oil	Soybean oil	Soybean oil	Salmon oil	Salmon oil	Salmon oil	ANOVA (P)		
Vitamin E (mg/kg)	25	250	2500	25	250	2500	Vitamin E	Oil	Vitamin E x oil
Gene^a^									
*ATF4*	1.00 ± 0.31	1.39 ± 0.28	1.06 ± 0.39	1.18 ± 0.39	1.19 ± 0.31	1.08 ± 0.27	0.065	0.99	0.16
*DDIT3*	1.00 ± 0.35	1.50 ± 0.63	1.33 ± 0.48	1.30 ± 0.39	1.47 ± 0.64	1.16 ± 0.36	0.074	0.79	0.28
*DNAJC3*	1.00 ± 0.12	1.18 ± 0.25	1.03 ± 0.23	1.09 ± 0.20	1.10 ± 0.19	0.98 ± 0.24	0.13	0.80	0.39
*EDEM1*	1.00 ± 0.16	1.11 ± 0.29	1.14 ± 0.21	0.86 ± 0.17	0.94 ± 0.19	0.90 ± 0.10	0.22	< 0.001	0.73
*HERPUD1*	1.00 ± 0.18	1.17 ± 0.33	1.10 ± 0.25	0.98 ± 0.32	1.15 ± 0.37	1.04 ± 0.27	0.18	0.67	0.97
*HSPA5*	1.00 ± 0.12	1.01 ± 0.22	0.95 ± 0.47	1.05 ± 0.22	1.17 ± 0.20	1.02 ± 0.21	0.24	0.059	0.62
*PDIA4*	1.00 ± 0.15	1.14 ± 0.26	1.01 ± 0.19	0.96 ± 0.18	1.05 ± 0.17	0.98 ± 0.20	0.12	0.27	0.86

Data are presented as means ± SD (*n* = 12/group)

^a^ *ATF4* activating transcription factor 4, *DDIT3* DNA damage inducible transcript 3, *DNAJC3* DnaJ heat shock protein family (Hsp40) member C3, *EDEM1* ER degradation enhancing alpha-mannosidase like protein 1, *HERPUD1* homocysteine inducible ER protein with ubiquitin like domain 1, *HSPA5* heat shock protein family A member 5, *PDIA4* protein disulfide isomerase family A, member 4

## Discussion

The present study was performed to investigate the effect of dietary vitamin E concentration on the Nrf2 regulated antioxidant and cytoprotective defense system. Therefore, we considered the liver of rats fed diets with a wide range of dietary vitamin E concentrations. The liver as the target tissue for investigation was selected for three reasons: (I) Due to its high metabolic activity, the liver is prone to a prooxidant condition which might interact with Nrf2 [38]; (II) Nrf2 plays a predominant role in the liver as it regulates not only antioxidant enzymes but also many enzymes involved in the hepatic detoxification system of phase I and phase II enzymes [39]; (III) tocopherol concentrations in the liver are strongly influenced by the dietary vitamin E concentration [40]. Based on the recommendations of vitamin E for rat diets (18 mg α-tocopherol equivalents/kg diet) by NRC [18], the lowest dietary vitamin E concentration (25 mg α-tocopherol equivalents/kg diet) used in this study might have been sufficient for growing rats. The concentrations of total tocopherols in the liver of rats receiving the diets with 25 mg vitamin E/kg (which were around 25 to 30 nmol/g) were lower than those observed in rats with adequate dietary vitamin E supply which are typically in the range between 50 and 100 nmol/g [27, 40, 41]. However, α-tocopherol concentrations in the liver of rats fed the diets with 25 mg vitamin E/kg in this study were much higher than in rats with strict vitamin E deficiency which were in most cases even below 2 nmol/g [27, 42]. Dietary vitamin E concentration of 250 and 2500 mg/kg diet are far in excess of the requirement of rats. However, these high concentrations could be representative for human beings using vitamin E as dietary supplement. As vitamins E is freely available in super markets or drug stores in high doses, the self-induced doses can reach levels from 500 to 1000 mg of vitamin E per day, equivalent to concentrations of 1000 to 2000 mg per kg diet dry matter intake. As expected, rats fed the diets with 2500 mg vitamin/kg showed a strong accumulation of α-tocopherol in the liver, in spite of the relatively short feeding period of four weeks. The finding that rats fed salmon oil had lower α-tocopherol concentrations in the liver at the high dietary vitamin E concentrations of 250 or 2500 mg/kg than those fed soybean oil agrees with studies in literature showing that high proportions of PUFA reduce concentrations of tocopherols in tissues and plasma. This effect is presumably due to the fact that incorporation of oxidation-susceptible PUFA into tissues causes more tocopherols to be consumed as antioxidants [43, 44].

We observed that the dietary vitamin E concentration did not influence feed intake and growth of the rats. This finding agrees with several other studies showing that the vitamin E supply over a wide range does not influence growth of rats [40, 45]. Even feeding a vitamin E-deficient diet containing only 2 mg of α-tocopherol per kg over the long period of 269 days did not impair growth of rats, indicating that even very low tissue vitamin E concentrations are uncritical with respect to growth [27]. Unexpectedly, rats fed the salmon oil diets showed a slightly higher feed intake which was accompanied with slightly higher body weight gains. The reason for this observation is unknown. However, as the feed:gain ratio was similar between the rats fed salmon oil and those fed soybean oil, it is assumed that there was no specific effect of the oil on growth of the rats.

In order to quantify ROS production, we used ESR spectroscopy with CMH as a spin probe molecule. Using this spin probe molecule, it is possible to detect superoxide anions, peroxy radicals, peroxynitrite and nitrogen dioxide [46]. We found that rats fed the fish oil diet with the lowest vitamin E showed the highest production of ROS in the liver which agrees with other studies showing that the combination of dietary fats with highly unsaturated fatty acids and a moderate dietary vitamin E concentration enhances the production of ROS by promoting lipid peroxidation [47–49]. As expected, an increase of dietary vitamin E concentrations led to a reduction of ROS production in the rats fed the salmon oil, due to an increased tocopherol concentration in the liver. Interestingly, increasing the dietary vitamin E concentrations of 250 mg vitamin E/kg to 2500 mg vitamin E/kg diet did not lead to a further reduction of radical production in the liver. This finding indicates that the dietary vitamin E concentration of 250 mg vitamin E/kg yielded maximum protection against lipid peroxidation in rats fed the salmon oil diet. In contrast to the groups of rats fed the salmon oil diet, minimum ROS production levels were even reached at the lowest dietary vitamin E concentration indicating that a dietary concentration of 25 mg of vitamin E per kg diet is sufficient to prevent against lipid peroxidation in rats fed soybean oil as a dietary fat source. The finding that rats fed the diets with 2500 mg vitamin E/kg diet did not produce a greater amount of radicals in the liver than rats fed the diets with 250 mg vitamin E/kg diet shows that excess vitamin E concentrations did not exert pro-oxidant effects in the liver of rats.

One of the main findings of this study is that an increased production of ROS in the liver, observed in the rats fed the fish oil diet with the lowest vitamin E concentration, did not cause a significant up-regulation of genes of the Nrf2 pathway and genes of the UPR in the liver of rats. This finding is in line with the observation that activities of hepatic antioxidant enzymes (GPX, SOD, CAT) which are targets of Nrf2, were not changed in the rats which showed an increased rate of ROS production. Our data therefore show that a moderate dietary oxidative stress induced by suboptimum dietary vitamin E does not induce significant activation of Nrf2 and induction of ER stress in the liver of rats. In the present study, we did not use vitamin E-deficient diets. Therefore, it remains unclear whether vitamin E deficiency, associated with the development of a stronger oxidative stress, could induce activation of Nrf2. In a previous study, it has been shown that severe vitamin E deficiency leads to an up-regulation of some enzymes involved in the protection against oxidative stress in muscle of rats, including heme oxygenase and GPX precursor, two enzymes regulated by Nrf2 [50]. These findings indicate that severe vitamin E deficiency could lead to an activation of Nrf2.

It has been suggested that high concentrations of dietary antioxidants, including vitamin E, could lead to a down-regulation of the endogenous defense system of the body [13, 17]. In the present study, we observed that feeding diets with a vitamin E concentration which is in 100-fold in excess in relation to the vitamin E requirement of rats did not cause a significant down regulation of Nrf2 genes and genes of the UPR, independent of the dietary oil used. In agreement with those findings, activities of antioxidant enzymes (GPX, SOD, CAT) were not reduced in rats fed the high vitamin E diet in comparison to rats fed the diets containing 25 or 250 mg vitamin E/kg. Therefore, our data suggest that an excess supply of vitamin E does not impair the antioxidant and cytoprotective defense system of the body. Nrf2 with its target genes involved in the antioxidant and cytoprotective system in the body has been recognized as an important longevity-promoting transcription factor in mammals [51]. The finding that vitamin E in the diet, from adequate to excess concentration, does not influence Nrf2 signaling is consistent with the view that vitamin E has no beneficial effect on the lifespan in various model organisms, including rat and mice [52].

With respect to the effects of vitamin E on Nrf2 signaling and ER stress, less studies are available so far. In a recent study, it has been shown that dietary vitamin E is able to cause an activation of Nrf2 in the aorta of hypercholesterolemic rabbits [53]. In another study, it has been found that α-tocopherol enhances the activation of Nrf2 in human retinal pigment epithelial cells subjected to oxidative stress by treatment with acrolein [54]. To our knowledge, no studies dealing with the effects of a broad range of dietary vitamin E concentration on Nrf2 and ER stress in the liver of animals have been published so far. There are however some studies which investigated the effects of dietary vitamin E concentrations on the activities of anti-oxidant enzymes in tissues of rats. These studies however revealed variable results. In the study of Shireen et al. [55], high concentrations of vitamin E in the diet (3680 mg/kg diet) increased activities of catalase, GPX and glutathione reductase in the liver of rats in comparison to a vitamin E-adequate control group. In the study of Ibrahim et al. [56], feeding a diet with 1000 mg vitamin E/kg did not influence the activities of SOD, CAT and GPX. In the study of Flader et al. [40], a vitamin E concentration of 3000 mg/kg diet lowered the activities of SOD and GPX in the liver of rats fed a diet with salmon oil, but not in rats fed a diet with lard as a source of fat. In Atlantic salmon, supplementation of the diet with 400 mg vitamin E/kg caused a strong reduction of lipid peroxidation but did not change activities of SOD and GPX in the liver [57]. A very recent study in rats investigated the effect of excess concentrations of vitamin E (100 and 200 mg/kg body weight) applied intraperitoneally for a period of either one or two weeks on activities of hepatic phase I and phase II enzymes, including several enzymes regulated by Nrf2 such as GST, CAT or NQO1 [58]. In that study, vitamin E application also did not substantially change activities of phase I and II enzymes in the liver. However, vitamin E application caused a down-regulation of some antioxidant enzymes in the kidney, suggesting that effects of vitamin E on Nrf2 target genes could be tissue-specific.

Previous in vitro-studies in micellar systems and low-density lipoproteins have demonstrated a pro-oxidative effect of high vitamin E concentrations [59–61]. This effect has been attributed to the formation of tocopheryl radicals which are able to react with PUFA yielding the production of peroxide radicals [61]. The present study shows that excess concentrations of vitamin E do not enhance the formation of radicals in vivo. It has been well established that tocopheryl radicals formed in vivo are scavenged by reaction with ascorbic acid, which might yield an explanation for this finding [62]. The finding that excess concentrations of vitamin E did not exert pro-oxidant effects agrees with some other at studies in which dietary vitamin E concentrations up to 10,000 mg/kg did not increase concentrations of lipid peroxidation products in the liver of rats [40, 54].

The experimental design of our study moreover allows to study the effect of fish oil as source of highly unsaturated n-3 PUFA in comparison to soybean oil on Nrf2 signaling and ER stress in the liver of rats. We observed that dietary salmon oil– independent of the dietary vitamin E concentration – increased mRNA concentrations of some of the Nrf2 target genes considered (*GCLM, NQO1, UGT1A1*). However, as concentrations of some Nrf2 target proteins (*GPX, HO1, NQO1*) as well as activities of antioxidant enzymes, all of which are targets of Nrf2, were not increased in rats fed the salmon oil diets in comparison to the rats fed the soybean oil diets, it is obvious that feeding salmon oil overall had less effect on Nrf2 pathway. The finding that relative mRNA concentrations of all of the UPR genes considered with the only exception of *EDEM1* were not different between rats fed salmon oil and those fed soybean oil, indicates that fish oil feeding did also not produce ER stress in the liver, independent of the dietary vitamin E concentration. *EDEM1* is an ER stress-induced gene which is targeted by XBP-1 [63]. EDEM1 overexpression studies have demonstrated a role in the degradation of misfolded proteins [64]. It is unclear why *EDEM1* was down-regulated in the rats fed the salmon oil whereas expression of *DNAJC3*, another gene controlled by XBP-1 was not influenced by the dietary oil. In any case, as the down regulation of EDEM1 in the rats fed salmon oil was only moderate (around 20% lower than in the group fed the salmon oil), the physiologic relevance of this effect might be limited.

To our knowledge, the effect of n-3 PUFA on Nrf2 and ER stress pathway in the liver of healthy rats has not yet been investigated. There are however some studies dealing with the effects of n-3 PUFA on Nrf2 signaling and ER stress in various cell models or in animal models with pathologic conditions. In liver and endothelial cells, treatment with n-3 PUFA (EPA, DHA) caused an activation of the Nrf2 pathway [65, 66]. The results of studies dealing with the impact of n-3 PUFA on ER stress are variable and are depending on the experimental conditions. There are some studies showing that n-3 PUFA are preventing against ER stress under experimental conditions which provoke ER stress such as in pancreatic cells treated with the ER stress inducer tunicamycin [67], in the brain of rats with traumatic brain injury [68] or in myoblasts which were stressed by statin treatment [69]. In opposite, there is also one study showing that fish oil supplementation in healthy human subjects induced genes of UPR in peripheral blood mononuclear cells [70]. In another study [71], DHA has been shown to induce ER stress in human cancer cells. These studies in overall suggest that n-3 PUFA might attenuate ER stress under experimental conditions in which ER stress has been induced. Our study shows that n-3 PUFA do not influence genes of the UPR under conditions with no or only moderate stress.

## Conclusion

In the present study, we investigated the effect of excess dietary vitamin E on the Nrf2 pathway in the liver of rats. This investigation is relevant for human health as many people are taking vitamin E supplements, some of them even in high doses, and Nrf2 as the master regulator of the cellular antioxidant and cytoprotective system is important for the prevention of several diseases including cancer, coronary heart disease or diabetes. The present study however shows that excess dietary vitamin E concentrations do not influence Nrf2 signaling, and also do not induce development of ER stress in the liver of rats. As the Nrf2 signaling pathway represents the most import antioxidant and cytoprotective system of the body, it is concluded that even very high vitamin E concentrations in the diet do not impair the endogenous defense system in the liver of healthy animals. Considering the group of rats fed the salmon oil diet, we found that dietary n-3 PUFA are uncritical with respect to the endogenous defense system and the production of ER stress.

## Abbreviations

ATF4: Activating transcription factor 4
ATP5B: ATP synthase
CANX: Calnexin
CAT: Catalase
CMH: 1-hydroxy-3-methoxycarbonyl-2,2,5,5-tetramethylpyrrolidine
DDIT3: DNA damage inducible transcript 3
DHA: Docosahexaenoic acid
DNAJC3: DnaJ heat shock protein family (Hsp40) member C3
EDEM1: ER degradation enhancing alpha-mannosidase like protein 1
EPA: Eicosapentaenoic acid
ER stress: Stress of the endoplasmic reticulum
ESR: Electron spin resonance spectroscopy
FAME: Fatty acid methyl esters
GCLM: Glutamate cysteine ligase, modifier subunit
GPX: Glutathione peroxidase 1
GSTA1: Glutathione S-transferase alpha 1
H +: Transporting, mitochondrial F1 complex, beta polypeptide
HERPUD1: Homocysteine inducible ER protein with ubiquitin like domain 1
HMOX1/HO-1: heme oxygenase 1
HSPA5: Heat shock protein family A member 5
KEAP1: Kelch like ECH associated protein
MDH1: Malate dehydrogenase 1
NFE2L2 (encoding Nrf2): Nuclear factor-erythroid 2-related factor-2
NQO1: NAD(P)H quinone dehydrogenase 1
PDIA4: Protein disulfide isomerase family A, member 4
ROS: Reactive oxygen species
SOD: Superoxide dismutase 1
SULT1B1: Sulfotransferase family 1B member 1
UGT1A6: UDP glucuronosyltransferase family 1 member A6
